# Supplementary material for: Virus-like particle (VLP)-based vaccine targeting tau phosphorylated at Ser396/Ser404 (PHF1) site outperforms phosphorylated S199/S202 (AT8) site in reducing tau pathology and restoring cognitive deficits in the rTg4510 mouse model of tauopathy
Source: Res Sq. 2024 Jun 12:rs.3.rs-4390998. Preprint. [Version 1] doi: 10.21203/rs.3.rs-4390998/v1 (PMC11213181; doi:10.21203/rs.3.rs-4390998/v1)
Supplement: 1 [file NIHPPrs4390998V1-supplement-1.pdf]

Supplemental material

Virus-like particle (VLP)-based vaccine targeting tau phosphorylated at Ser396/Ser404 (PHF1) site outperforms phosphorylated S199/S202 (AT8) site in reducing tau pathology and restoring cognitive deficits in the rTg4510 mouse model of tauopathy

Jonathan Hulse[1, †], Nicole Maphis[2, †], Julianne Peabody[1], Bryce Chackerian[1], Kiran Bhaskar[*1,3]
[1]Department of Molecular Genetics & Microbiology, [2]Department of Neurosciences, [3]Department of Neurology, University Of New Mexico, Albuquerque, NM. USA.

[†]Authors contributed equally and share first-authorship.
*Correspondence:
Kiran Bhaskar, Ph.D.
MSC08 4660, 1 University of New Mexico
Albuquerque NM 87131, USA.
kbhaskar@salud.unm.edu
+1-505-272-1230

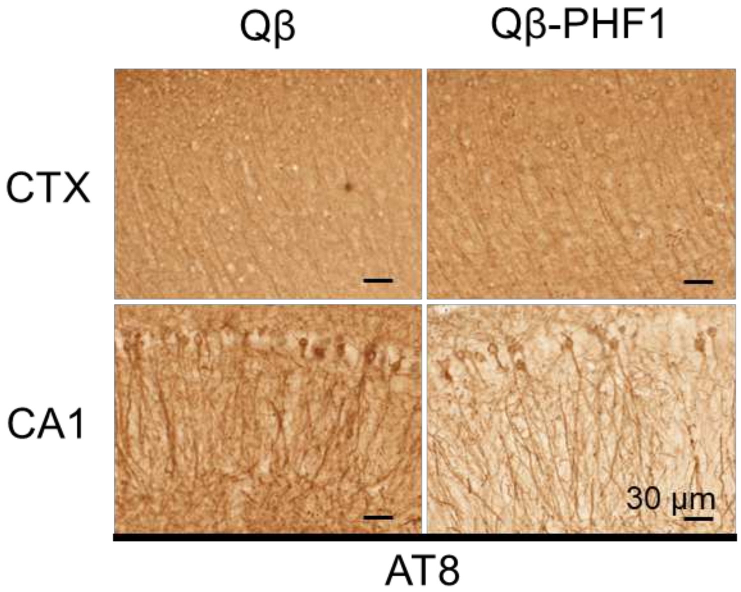

Supplementary Fig S1. Qβ-PHF1 vaccination fails to reduce AT8 pathology in rTg4510 mice. IHC of brain sections from Qβ Control and Qβ-PHF1 vaccinated rTg4510 mice using AT8 was performed to validate the observations from Western blot analysis that soluble AT8 levels are unchanged in Qβ-PHF1 vaccinated mice compared to Qβ Controls. No obvious differences are observed in AT8 histopathology between Qβ Control and Qβ-PHF1 vaccinated rTg4510 mice in either the cerebral cortex or CA1 hippocampus.

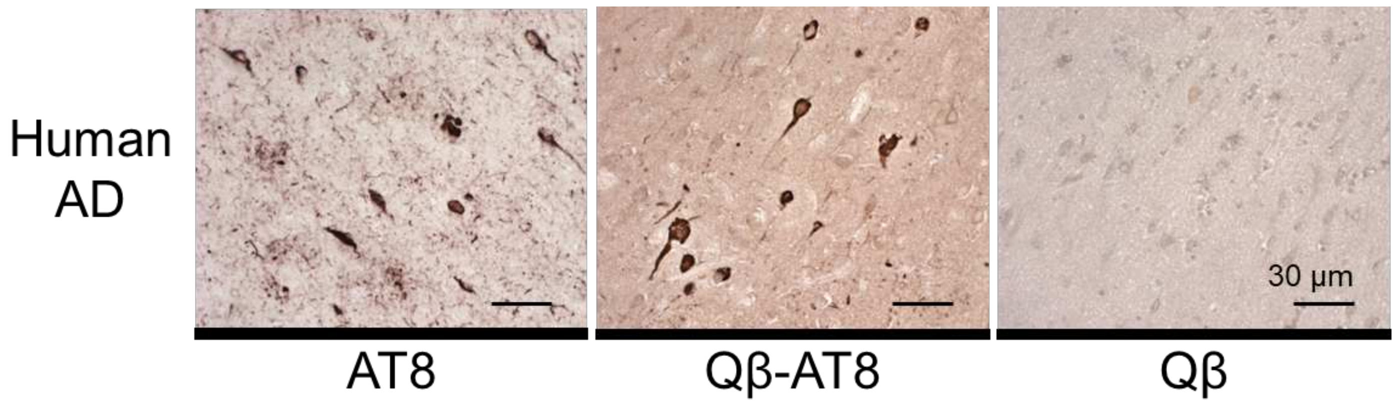

Supplementary Fig S2. Immune sera from Qβ Control and Qβ-AT8 vaccinated mice was used to stain human post-mortem hippocampal sections from AD brain tissue and compared to AT8 histopathology. Qβ-AT8 immune sera stained somatodendritic NFT and ghost tangle histopathology in AD brain tissue similarly to AT8 but did not robustly stain other pathological features observed in AT8 such as neuropil threads or neuritic plaques. Qβ Control sera did not exhibit specific staining of any tau pathology.
